# Early Diagnosis and Successful Treatment of Influenza Coinfection With Community‐Onset Methicillin‐Resistant *Staphylococcus aureus* Necrotizing Pneumonia: A Case Report

**DOI:** 10.1155/crdi/8950164

**Published:** 2026-08-05

**Authors:** Po-Kai Chan, Chun-Han Wu

**Affiliations:** ^1^ Department of Medicine, Tri-Service General Hospital, National Defense Medical Center, Taipei, Taiwan, ndmctsgh.edu.tw; ^2^ Division of Cardiology, Department of Medicine, Tri-Service General Hospital, National Defense Medical Center, Taipei, Taiwan, ndmctsgh.edu.tw; ^3^ Division of Pulmonary and Critical Care Medicine, Department of Medicine, Tri-Service General Hospital, National Defense Medical Center, Taipei, Taiwan, ndmctsgh.edu.tw

**Keywords:** case report, community-acquired pneumonia, influenza A, MRSA, necrotizing pneumonia, RT-PCR

## Abstract

**Background:**

Necrotizing pneumonia is a severe and potentially fatal complication of community‐acquired pneumonia, often associated with toxin‐producing or drug‐resistant pathogens. Rapid and accurate identification of these pathogens is crucial for timely intervention. Polymerase chain reaction (PCR)–based diagnostic tools, such as the BioFire FilmArray pneumonia panel, have significantly improved early pathogen detection, aiding in prompt and targeted treatment as in the presenting case.

**Case Report:**

We report a case of necrotizing pneumonia in a female adult who presented with severe respiratory distress. Initial testing identified coinfection with influenza and methicillin‐resistant *Staphylococcus aureus* (MRSA) using the BioFire FilmArray pneumonia panel, which provided rapid and precise pathogen detection before admission. The patient developed worsening respiratory failure, requiring mechanical ventilation and intensive care. Despite the severity of the infection, early diagnosis and appropriate antimicrobial therapy tailored to the identified pathogens led to a significant clinical improvement, allowing for a favorable recovery.

**Conclusion:**

This case highlights the critical role of rapid molecular diagnostics in the early detection of coinfections in necrotizing pneumonia. The timely identification of influenza and MRSA facilitated targeted antimicrobial therapy, which was instrumental in preventing further complications and improving the patient’s prognosis. As PCR‐based diagnostics become more widely available, their integration into routine clinical practice can enhance the management of severe pneumonia cases, ultimately leading to better outcomes. Clinicians should maintain a high index of suspicion for coinfections in severe pneumonia and leverage rapid diagnostic tools to guide early and effective treatment strategies.

## 1. Introduction

Necrotizing pneumonia stands as a severe complication of community‐onset pneumonia, carrying a high mortality rate, particularly when caused by methicillin‐resistant *Staphylococcus aureus* (MRSA) following influenza infection. Although coinfection of influenza and MRSA is a recognized clinical entity, its diagnosis in the early phase of presentation remains challenging, as conventional cultures typically require 48–72 h, and clinicians must rely on empirical broad‐spectrum coverage during this critical window. The polymerase chain reaction (PCR) offers a rapid and valuable tool for identifying specific toxin‐encoding or drug‐resistant pathogens. Herein, we report a case of necrotizing pneumonia with coinfection of influenza and MRSA, in which the BioFire FilmArray pneumonia panel was deployed at the ED within hours of presentation. This case illustrates how front‐door rapid syndromic testing can translate a conditional guideline recommendation into actionable, pathogen‐confirmed therapy and serves as a practical model for diagnostic stewardship in critically ill patients with suspected severe pulmonary coinfection.

## 2. Case Report

**FIGURE 1:**
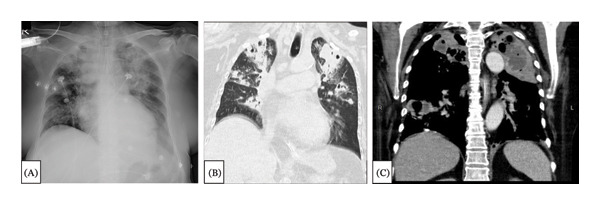
The chest X‐ray on Day 1 showed multifocal ground glass opacities (GGOs) and consolidation over bilateral lungs (A), while the computer tomography (CT) without contrast additionally showed few possible cavitary foci (B). The following CT with contrast on Day 15 showed mild resolution of GGOs yet progressing cavitary foci and air fluid levels (C), suggesting a lung abscess and necrotizing pneumonia formation.

A 65‐year‐old female with a history of Type 2 diabetes and hypertension suffered from high fever, productive cough, and shortness of breath for 2 days. She visited the local regional hospital, where the chest radiography (CXR, Figure 1(A)) showed multiple patch opacities over bilateral lung fields, which was compatible with pneumonia. Due to respiratory pattern deterioration with accessory muscle use and severe hypoxemia, she was transferred to our hospital, a tertiary medical center, for further care.

At our emergency department (ED), the physical examination revealed a body temperature of 38.9°C, heart rate of 99 beats/minute, respiratory rate of 20 breaths/minute, blood pressure of 87/60 mmHg, and oxygen saturation of 74% under a nonrebreathing mask with an 80% fraction of inspired oxygen (FiO2) support. The blood gas analysis showed an arterial partial pressure of oxygen (PaO2) 66 mmHg. Because of impending respiratory failure, endotracheal intubation was performed with mechanical ventilator support. Laboratory tests revealed leukocytosis with increased neutrophils (92%), extremely high C‐reactive protein (CRP, > 48 mg/dL) level, deteriorated serum creatinine 3.2 mg/dL, and lactate 2.6 mg/dL. A chest computed tomography (CT) without contrast showed multifocal peribronchial consolidations and ground glass opacities (GGOs) in all pulmonary lobes with few possible cavitary foci (Figure 1(B)). Under the impression of community‐acquired pneumonia with septic shock and acute respiratory failure, the patient received aggressive crystalloid fluid resuscitation with high‐dose norepinephrine infusion and empirical antibiotics with meropenem and clarithromycin.

On Day 2 at the ED, the real‐time PCR (RT‐PCR) testing of sputum, using the BioFire FilmArray® pneumonia panel (BioFire Diagnostics, LLC, Salt Lake City, UT), collected at the ED yielded a positive result of Type A influenza virus and *Staphylococcus aureus* with mecA/C and mec right‐extremity junction (MREJ) gene mutation, indicating MRSA infection. Oseltamivir was initiated for influenza, and we shifted the antibiotics to teicoplanin with meropenem for MRSA. On Day 3, she was admitted to our intensive care unit (ICU), while the endotracheal‐aspirated sputum culture and the first blood culture collected at the ED both yielded MRSA. Antibiotic sensitivity displayed that the organism was resistant to penicillin, levofloxacin, and erythromycin; moderately sensitive to moxifloxacin; and sensitive to vancomycin, teicoplanin, daptomycin, and linezolid (Table 1). The diagnosis of coinfection with influenza A and MRSA pneumonia, complicated by bacteremia and septic shock, was established. We continued the oseltamivir in addition to antibiotics with teicoplanin and meropenem based on sensitivity testing.

**TABLE 1:** Antimicrobial susceptible test in strain isolated from the patient.

Antimicrobial agent	Susceptibility	MIC
Penicillin‐G	Resistant	≥ 0.5 mg/L
Oxacillin	Resistant	≥ 4 mg/L
Clindamycin	Susceptible	0.3 μg/mL
Erythromycin	Resistant	≥ 8 mg/L
Gentamicin	Susceptible	≤ 0.5 mg/L
Sulfamethoxazole/trimethoprim	Susceptible	≤ 10 mg/L
Rifampin	Susceptible	≤ 0.5 mg/L
Tigecycline	Susceptible	≤ 0.12 mg/L
Moxifloxacin	Intermediate	1.0 μg/mL
Levofloxacin	Resistant	4.0 mg/mL
Ceftaroline	Susceptible	0.5 μg/mL
Vancomycin	Susceptible	1.0 μg/mL
Linezolid	Susceptible	2.0 μg/mL
Teicoplanin	Susceptible	≤ 0.5 mg/L
Daptomycin	Susceptible	0.3 μg/mL

**FIGURE 2:**
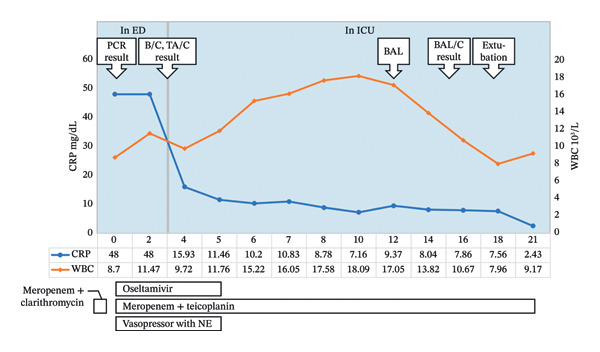
Clinical course and laboratory trends of this patient. The *x*‐axis indicated the timeline days after the emergency department visit. The blue line represents the trend of CRP levels. The orange line indicates WBC levels. Antibiotics shifted from clarithromycin to teicoplanin once the PCR report revealed gene mutations indicating MRSA infection, which was later confirmed in bacterial cultures. The CRP level dropped significantly, while the WBC levels peaked before the bronchoscopy on Day 12. *Abbreviations:* B/C, blood culture; BAL, bronchoalveolar lavage; CRP, C‐reactive protein; WBC, white blood cells; TA/C, endotracheal‐aspirated sputum culture; PCR, real‐time polymerase chain reaction; NE, norepinephrine.

During the period of hospitalization, the series of CXRs revealed persistent GGOs with multiple cavitary lesions over bilateral lung fields. The following laboratory data showed a rapid decline in CRP level, while leukocytosis persisted (Figure 2). On Day 12, due to poor resolution of GGO, bronchoscopy was performed with aspiration of purulent sputum and bronchoalveolar lavage (BAL). The result of the BAL specimens also confirmed the presence of MRSA infection with identical sensitivity results as previous culture data in serum and sputum aspirated from the endotracheal tube. Subsequent chest CT scans with contrast on Day 15 demonstrated multifocal peribronchial consolidations and cavitary foci with air‐fluid levels in all pulmonary lobes, indicating necrotizing pneumonia and lung abscesses (Figure 1(C)). Despite limited resolution on image findings, the patient’s respiratory condition and oxygenation improved after the bronchoscopy, and she was successfully extubated on Day 17 and was transferred to the regular ward on Day 21. The patient expressed gratitude for the prompt diagnosis and treatment. Although the recovery process was challenging, the patient felt reassured by the medical team’s clear communication and the targeted treatment approach. Under stable conditions, the patient was transferred to the local regional hospital on Day 28 and received 6 weeks of antibiotic therapy.

## 3. Discussion

Numerous studies have suggested increasing secondary or coinfection with MRSA during influenza season [1, 2]. Compared to hospital‐acquired MRSA, several reports describe rapid progression of community‐acquired MRSA pneumonia with severe complications such as pleural empyema, necrotizing pneumonia, or pneumothorax in both children and adults, contributing to a significantly high mortality rate [3, 4]. Of note, distinguishing community‐associated MRSA (CA‐MRSA) from hospital‐associated MRSA (HA‐MRSA) based solely on clinical presentation in the acute setting remains challenging, as detailed epidemiological history (prior hospitalization, indwelling devices, and healthcare exposure) is often unavailable or unreliable at initial presentation. We therefore adopt the term “community‐onset MRSA” to describe our case, acknowledging that molecular typing (e.g., SCCmec type and PVL gene detection) would be required for definitive classification but was beyond the scope of our diagnostic resources. In our care, early recognition of the pathogen with the help of RT‐PCR at the ED led to the accurate choice of antibiotics and favorable outcome.

The interplay between influenza virus and MRSA in the context of coinfection remains an area of interest. There are several hypothetical mechanisms for coinfection. First, the influenza virus may weaken the immune response and increase the adhesion of bacteria by destroying the epithelial layer of the tracheobronchial tree and neuraminidase activity, making individuals more susceptible to MRSA infection [5]. Second, bacterial factors may contribute to the coinfection and disease severity. Pore‐forming toxins such as leukocidin AB (LukAB) and Panton–Valentine leucocidin (PVL) produced by community‐acquired MRSA have been suggested as potential contributors to this phenomenon. LukAB binds to soluble heparan sulfate (HS) sequences produced by host cells, augments cytotoxicity both in vitro and in vivo, and interferes with cell signaling, growth, motility, and differentiation as well as the response to infection, leading to lung injury and immune infiltration in the post influenza infection lung microenvironment [5]. Moreover, PVL increases cell permeability and loss of potassium ions and induces a strong lytic effect on host defense cells. Previously infected influenza virus can enhance the proinflammatory and cytotoxic effects of PVL on neutrophils, causing pulmonary tissue damage and developing necrotizing pneumonia [6].

With an understanding of pathophysiology, several treatment options were discussed in treating MRSA. Antimicrobials that impede protein synthesis and consequently reduce toxin production, such as high‐dose clindamycin and linezolid, have been recommended for countering toxin‐related pathologies [1]. However, linezolid is not usually advocated for MRSA bacteremia due to adverse effects of bone marrow suppression. Teicoplanin, despite a potential need for dose escalation in severe cases, can be an alternative option for MRSA pneumonia with similar efficacy yet fewer adverse effects compared to vancomycin and linezolid [7, 8]. In our case, teicoplanin was specifically selected over vancomycin given the patient’s deteriorated renal function on admission (serum creatinine 3.2 mg/dL) and the favorable nephrotoxicity profile of teicoplanin, in accordance with our institutional guidelines for severe MRSA pneumonia in patients with acute kidney injury. In addition, early adjuvant immunoglobulins in single or dual doses are recommended for severe and critical diseases due to their potential to neutralize PVL toxins, despite limited clinical trial evidence [9]. With early identification of MRSA at ED and appropriate treatment, ventilator requirements and ICU stays were shortened.

A key learning point from this case lies not in the coinfection entity itself—which is well documented—but in the timing and clinical impact of rapid syndromic testing deployed at the ED rather than after ICU admission or treatment failure. The 2023 ERS/ESICM/ESCMID/ALAT guidelines for severe community‐acquired pneumonia (sCAP) conditionally recommend sending lower respiratory tract specimens for multiplex PCR testing whenever nonstandard sCAP antibiotics are prescribed or considered, though the supporting evidence is graded as very low quality [10]. Our case provides granular, real‐world evidence supporting the implementation of this recommendation at the very front door of the hospital. While empirical anti‐MRSA coverage remains the appropriate clinical decision for necrotizing pneumonia following influenza, empiricism is, by definition, an estimation. The BioFire FilmArray pneumonia PCR panel, an FDA‐cleared sample‐to‐answer assay, has been proven to be highly sensitive in detecting bacterial pathogens and antimicrobial resistance genes from lower respiratory tract specimens [11]. Preadmission deployment of this assay enabled us to transition from “suspecting MRSA” to “confirming MRSA with mecA/MREJ resistance markers” within hours of presentation, allowing immediate, precision‐guided therapy rather than prolonged broad‐spectrum coverage. This reflects a meaningful shift from empirical coverage to diagnostic‐driven therapy and represents a practical model of antimicrobial and diagnostic stewardship in the acute care setting.

Interpreting multiplex PCR results in the clinical context remains essential, as the panel may detect organisms that represent colonization rather than active infection. In our case, the FilmArray panel additionally detected *Haemophilus influenzae* (≥ 10^7^ copies/mL, Table 2) and a non–SARS‐CoV‐2 coronavirus alongside influenza A and MRSA. We interpreted these additional findings as likely colonization or nonprimary drivers of the necrotizing pneumonia, based on three considerations: (1) the radiographic and clinical phenotype—multifocal cavitation with air‐fluid levels—is highly characteristic of staphylococcal necrotizing pneumonia rather than *H. influenzae* or viral pneumonitis; (2) the semiquantitative copy numbers and clinical trajectory did not support an alternative primary pathogen; and (3) effective coverage for MRSA and influenza was already provided by teicoplanin and oseltamivir, with meropenem additionally covering *H. influenzae*. This illustrates an inherent limitation of PCR‐based syndromic panels: They cannot reliably distinguish infection from carriage, and results must always be integrated with clinical, radiographic, and microbiological context. Nonetheless, the rapid identification of mecA‐encoding MRSA was decisive in guiding early targeted therapy in our case.

**TABLE 2:** The BioFire FilmArray pneumonia panel result.

Pathogen or gene	Result
*Bacteria*	
ACB complex	Not detected.
*K. aerogenes*	Not detected.
*E. cloacae* cpx	Not detected.
*E. coli*	Not detected.
*H. influenzae*	Detected.
*H. influenzae* Bin	≥ 10^7 copies/mL
*K. oxytoca*	Not detected.
*K. pneumoniae*	Not detected.
*M. catarrhalis*	Not detected.
*Proteus spp*.	Not detected.
*P. aeruginosa*	Not detected.
*S. marcescens*	Not detected.
*S. aureus*	Detected.
*S. aureus* Bin	≥ 10^7 copies/mL
*S. agalactiae*	Not detected.
*S. pneumoniae*	Not detected.
*S. pyogenes*	Not detected.

*Antimicrobial resistance genes*	
mecA/C and MREJ (FAPN)	Detected.
KPC (FAPN)	Not detected.
NDM (FAPN)	Not detected.
Oxa‐48‐like (FAPN)	Not detected.
VIM (FAPN)	Not detected.
IMP (FAPN)	Not detected.
CTX‐M (FAPN)	Not detected.

*Atypical pathogen*	
*Chlamydia p*.	Not detected.
*Legionella p*.	Not detected.
*Mycoplasma p.*	Not detected.

*Virus*	
Adenovirus	Not detected.
Coronavirus (not SARS‐CoV‐2)	Detected.
Metapneumovirus	Not detected.
Rhino/Enterovirus	Not detected.
Influenza A	Detected.
Influenza B	Not detected.
Parainfluenza	Not detected.
RSV	Not detected.

A major limitation of this report was the inability to confirm the presence of the PVL toxin due to limited laboratory resources, which would have further characterized the virulence profile of the isolate. In addition, formal SCCmec typing to definitively classify CA‐MRSA versus HA‐MRSA was not performed. Despite these limitations, the favorable outcome achieved through early rapid diagnostics supports the broader implementation of multiplex PCR at the ED level in cases of suspected severe coinfection.

## 4. Conclusion

This case illustrates that the clinical value of multiplex PCR in severe community‐onset pneumonia depends not only on its availability but also on where it is deployed. By performing the BioFire FilmArray Pneumonia Panel at the ED rather than after ICU admission, we transitioned from empirical broad‐spectrum coverage to pathogen‐confirmed therapy within hours, enabling timely administration of teicoplanin and oseltamivir for confirmed MRSA and influenza A coinfection while avoiding overtreatment for likely colonizers. Real‐world cases such as ours provide practical support for elevating the conditional ERS/ESICM/ESCMID/ALAT recommendation for rapid syndromic testing into a front‐door diagnostic standard—a meaningful step from empirical to precision‐guided antimicrobial stewardship in critically ill patients.

## Author Contributions

Po‐Kai Chan wrote the first draft of the manuscript and performed the extraction of the data, and Chun‐Han Wu reviewed the manuscript.

## Funding

This work did not receive specific funding.

## Disclosure

All authors read and approved the final manuscript.

## Ethics Statement

Ethical approval for the study was obtained from the Institutional Review Board of Tri‐Service General Hospital (TSGHIRB B‐2023‐15‐174).

## Consent

Written informed consent was obtained from all patients prior to their participation.

## Conflicts of Interest

The authors declare no conflicts of interest.

## Supporting Information

Additional supporting information can be found online in the Supporting Information section.

Each item on the checklist has been addressed within the manuscript, with specific references to the relevant section or paragraph, ensuring that the case report meets the standardized criteria for high‐quality reporting.

## Supporting information


**Supporting Information 1** The supporting information includes the CAse REport (CARE) checklist, which outlines the essential items recommended for inclusion in a case report. The checklist is based on the CARE guidelines, developed to improve the transparency and completeness of case report reporting.

## Data Availability

The data that support the findings of this study are available on request from the corresponding author. The data are not publicly available due to privacy or ethical restrictions.
