# Supplementary material for: Early Diagnosis and Successful Treatment of Influenza Coinfection With Community‐Onset Methicillin‐Resistant Staphylococcus aureus Necrotizing Pneumonia: A Case Report
Source: Case Rep Infect Dis. 2026 Aug 5;2026:8950164. doi: 10.1155/crdi/8950164 (PMC13439225; doi:10.1155/crdi/8950164)
Supplement: Supplementary file 1 [file CRDI-2026-8950164-s001.pdf]

CARE case report guidelines

CARE Checklist of information to include when writing a case report

(cc) BY-NC-ND

Topic	Item	Checklist item description	Reported on Line
Title	1	The diagnosis or intervention of primary focus followed by the words "case report"	Page1, Line 4
Key Words	2	2 to 5 key words that identify diagnoses or interventions in this case report, including "case report"	Page1, Line 4
Abstract (no references)	3a	Introduction: What is unique about this case and what does it add to the scientific literature?	Page2, Line 5
	3b	Main symptoms and/or important clinical findings	Page2, Line 9
	3c	The main diagnoses, therapeutic interventions, and outcomes	Page2, Line 9
	3d	Conclusion—What is the main "take-away" lesson(s) from this case?	Page2, Line 18
Introduction	4	One or two paragraphs summarizing why this case is unique (**may include references**)	Page3, Line 3
Patient Information	5a	De-identified patient specific information.	V
	5b	Primary concerns and symptoms of the patient.	Page4, Line 3
	5c	Medical, family, and psycho-social history including relevant genetic information	Page4, Line 2
	5d	Relevant past interventions with outcomes	X
Clinical Findings	6	Describe significant physical examition (PE) and important clinical findings.	Page4, Line10
Timeline	7	Historical and current information from this episode of care organized as a timeline	V
Diagnostic Assessment	8a	Diagnostic testing (such as PE, laboratory testing, imaging, surveys).	Page4, Line12
	8b	Diagnostic challenges (such as access to testing, financial, or cultural)	X
	8c	Diagnosis (including other diagnoses considered)	Page5, Line5
	8d	Prognosis (such as staging in oncology) where applicable	X
Therapeutic Intervention	9a	Types of therapeutic intervention (such as pharmacologic, surgical, preventive, self-care)	Page5, Line7
	9b	Administration of therapeutic intervention (such as dosage, strength, duration)	Page5, Line24
	9c	Changes in therapeutic intervention (with ratiole)	X
Follow-up and Outcomes	10a	Clinician and patient-assessed outcomes (if available)	X
	10b	Important follow-up diagnostic and other test results	Page5, Line9, 15
	10c	Intervention adherence and tolerability (How was this assessed?)	Page4, Line10
	10d	Adverse and unticipated events	X
Discussion	11a	A scientific discussion of the strengths AND limitations associated with this case report	Page6, Line 13
	11b	Discussion of the relevant medical literature **with references**.	V
	11c	The scientific rationale for any conclusions (including assessment of possible causes)	V
	11d	The primary "take-away" lessons of this case report (without references) in a one paragraph conclusion	Page5. Line10
Patient Perspective	12	The patient should share their perspective in one to two paragraphs on the treatment(s) they received	Page7, Line 20
Informed Consent	13	Did the patient give informed consent? Please provide if requested	**Yes** ☑ **No** ☐
